# 1-{3-(4-Methyl­phen­yl)-5-[5-(2-nitro­phen­yl)furan-2-yl]-4,5-di­hydro-1*H*-pyrazol-1-yl}ethanone

**DOI:** 10.1107/S1600536813024690

**Published:** 2013-09-12

**Authors:** N. Vinutha, S. Madan Kumar, B. S. Vidyashree Jois, Kalluraya Balakrishna, N. K. Lokanath, D. Revannasiddaiah

**Affiliations:** a Department of Studies in Physics, University of Mysore, Manasagangotri, Mysore 570 006, India; b Department of Studies in Chemistry, Mangalore University, Mangalagangotri, Mangalore 574 199, India

## Abstract

In the title compound, C_22_H_19_N_3_O_4_, the dihedral angle between the furan and pyrazole rings is 82.73 (19)° while the dihedral angles between the furan and pyrazole rings and their attached benzene rings are 31.93 (18) and 1.88 (18)°, respectively. In the crystal, inversion dimers linked by pairs of C—H⋯O hydrogen bonds generate *R*_2_^2^(16) loops. In addition, weak C—H⋯π and aromatic π–π stacking [minimum centroid–centroid distance = 3.5374 (17) Å] inter­actions are observed.

## Related literature

For background to the biological properties of pyrazole derivatives, see: Amir *et al.* (2008 ▶); Husain *et al.* (2008 ▶).
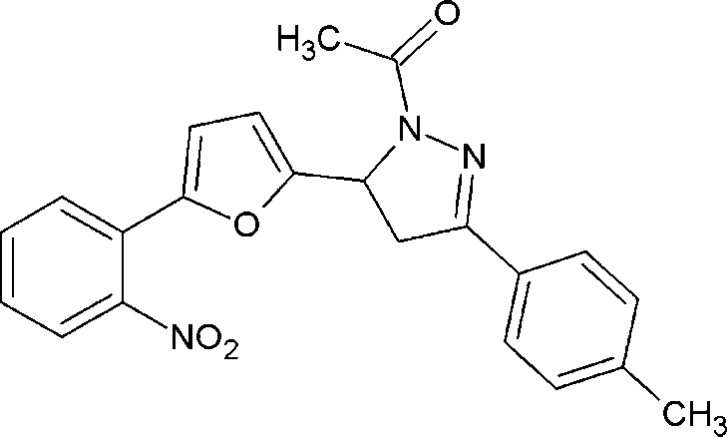


## Experimental

### 

#### Crystal data

C_22_H_19_N_3_O_4_
*M* *_r_* = 389.40
Triclinic, 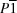

*a* = 7.6235 (3) Å
*b* = 10.5652 (4) Å
*c* = 13.1177 (4) Å
α = 103.344 (2)°
β = 95.025 (2)°
γ = 108.221 (2)°
*V* = 961.78 (6) Å^3^
*Z* = 2
Cu *K*α radiation
μ = 0.78 mm^−1^
*T* = 296 K
0.23 × 0.22 × 0.21 mm

#### Data collection

Bruker X8 Proteum CCD diffractometer
Absorption correction: multi-scan (*SADABS*; Bruker, 2013 ▶) *T*_min_ = 0.842, *T*_max_ = 0.854
10640 measured reflections
3083 independent reflections
2186 reflections with *I* > 2σ(*I*)
*R*_int_ = 0.138

#### Refinement

*R*[*F* ^2^ > 2σ(*F* ^2^)] = 0.084
*wR*(*F* ^2^) = 0.242
*S* = 1.06
3083 reflections
265 parameters
H-atom parameters constrained
Δρ_max_ = 0.40 e Å^−3^
Δρ_min_ = −0.38 e Å^−3^

### 

Data collection: *APEX2* (Bruker, 2013 ▶); cell refinement: *SAINT* (Bruker, 2013 ▶); data reduction: *SAINT*; program(s) used to solve structure: *SHELXS97* (Sheldrick, 2008 ▶); program(s) used to refine structure: *SHELXL97* (Sheldrick, 2008 ▶); molecular graphics: *Mercury* (Macrae *et al.*, 2008 ▶); software used to prepare material for publication: *SHELXL97*.

## Supplementary Material

Crystal structure: contains datablock(s) global, I. DOI: 10.1107/S1600536813024690/hb7133sup1.cif

Structure factors: contains datablock(s) I. DOI: 10.1107/S1600536813024690/hb7133Isup2.hkl

Supplementary material file. DOI: 10.1107/S1600536813024690/hb7133Isup3.cml

Additional supplementary materials:  crystallographic information; 3D view; checkCIF report

## Figures and Tables

**Table 1:** Hydrogen-bond geometry (Å, °) *Cg*4 is the centroid of the C23–C28 ring.

*D*—H⋯*A*	*D*—H	H⋯*A*	*D*⋯*A*	*D*—H⋯*A*
C14—H14⋯O19^i^	0.93	2.56	3.462 (4)	162
C18—H18*C*⋯*Cg*4^ii^	0.96	2.69	3.595 (4)	158

Symmetry codes: (i) 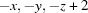; (ii) 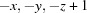.

**Fig. 1.**
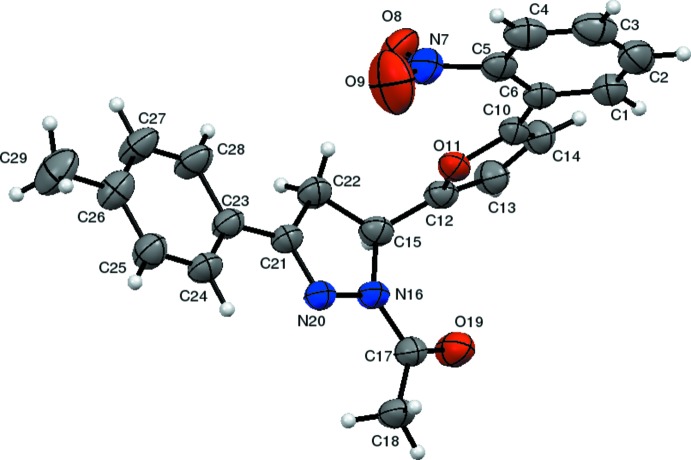
ORTEP diagram of the title compound with 50% probability ellipsoids.

**Fig. 2.**
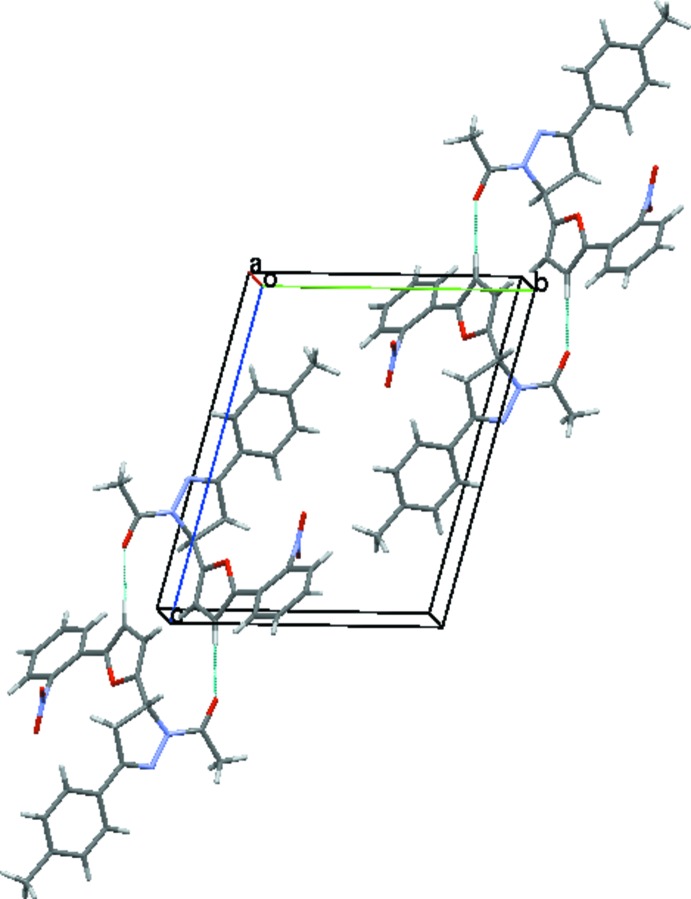
Packing diagram of molecule, viewed along the crystallographic a axis. Dotted lines represent hydrogen bonds.

## supplementary crystallographic information

### 1. Comment

The ongoing work in our lab for synthesizing pyrozoline derivatives resulted the title molecule. Similar compounds are used for the preparation of drugs in the pharmacological industries (Amir *et al.*, 2008; Husain *et al.*, 2008).

In the title compound (Fig. 1), the pyrazole ring makes a dihedral angle of 82.73 (19) °, 88.84 (18) ° and 1.88 (18) ° with furan, nitrophenyl and terminal methylphenyl rings, respectively. The furan ring makes a dihedral angle of 82.35 (18) ° and 31.93 (18) ° with methyl phenyl and nitrophenyl rings, respectively. The dihedral angle between terminal nitrophenyl and methylphenyl ring is 88.23 (17) °.

The title molecules are linked to one another with C14—H14···O19 intermolecular hydrogen bonds with *R*_2_^2^(16) ring motif (Fig. 2 and Table 1). In addition, short contacts C—H···π (*Cg*4) with distance 3.595 (4) Å (angle 158 °) [-*x*, -*y*, *z* - 1]. And π···π between *Cg*2 and *Cg*1 with distance 3.5374 (17) ° [*x*,*y*,*z*] and between *Cg*2 and *Cg*4 with a distance 3.641 (2) ° [*x* - 1, *-y*, *z* - 1] were observed, where *Cg*1:O11/C10/C14/C13/C12, *Cg*2:N16/N20/C21/C22/C15 and *Cg*4:C23/C24/C25/C26/C27/C28.

### 2. Experimental

A mixture of 4-methylphenyl-3-[5-(2-nitrophenyl)furan-2-yl]prop-2-en-1-one (10 mmol), hydrazine hydrate (50 mmol) and glacial acetic acid (40 ml) were refluxed for 24 h. The resulting mixture was poured into water (100 ml) and allowed to stand. The precipitate that formed was separated by filtration, washed with cold water and then recrystallized from mixed DMF-ethanol solvents to yield brown blocks.

### 3. Refinement

All the H atoms were fixed geometrically (C—H= 0.93–0.96 Å) and allowed to ride on their parent atoms with *U*_iso_(H) =1.5*U*_eq_(*C*-methyl) and = 1.2*U*_eq_(C) for other H atoms.

### Crystal data

**Table:**

C_22_H_19_N_3_O_4_	*Z* = 2
*M**_r_* = 389.40	*F*(000) = 408
Triclinic, *P*1	*D*_x_ = 1.345 Mg m^−^^3^
Hall symbol: -P 1	Cu *K*α radiation, λ = 1.54178 Å
*a* = 7.6235 (3) Å	Cell parameters from 3083 reflections
*b* = 10.5652 (4) Å	θ = 3.5–64.8°
*c* = 13.1177 (4) Å	µ = 0.78 mm^−^^1^
α = 103.344 (2)°	*T* = 296 K
β = 95.025 (2)°	Block, brown
γ = 108.221 (2)°	0.23 × 0.22 × 0.21 mm
*V* = 961.78 (6) Å^3^	

### Data collection

**Table:**

Bruker X8 Proteum CCD diffractometer	3083 independent reflections
Radiation source: Bruker MicroStar microfocus rotating anode	2186 reflections with *I* > 2σ(*I*)
Helios multilayer optics monochromator	*R*_int_ = 0.138
Detector resolution: 10.7 pixels mm^-1^	θ_max_ = 64.8°, θ_min_ = 3.5°
φ and ω scans	*h* = −8→8
Absorption correction: multi-scan (*SADABS*; Bruker, 2013)	*k* = −12→12
*T*_min_ = 0.842, *T*_max_ = 0.854	*l* = −14→15
10640 measured reflections	

### Refinement

**Table:**

Refinement on *F*^2^	Secondary atom site location: difference Fourier map
Least-squares matrix: full	Hydrogen site location: inferred from neighbouring sites
*R*[*F*^2^ > 2σ(*F*^2^)] = 0.084	H-atom parameters constrained
*wR*(*F*^2^) = 0.242	*w* = 1/[σ^2^(*F*_o_^2^) + (0.1607*P*)^2^] where *P* = (*F*_o_^2^ + 2*F*_c_^2^)/3
*S* = 1.06	(Δ/σ)_max_ < 0.001
3083 reflections	Δρ_max_ = 0.40 e Å^−^^3^
265 parameters	Δρ_min_ = −0.38 e Å^−^^3^
0 restraints	Extinction correction: *SHELXL97* (Sheldrick, 2008), FC^*^=KFC[1+0.001XFC^2^Λ^3^/SIN(2Θ)]^-1/4^
Primary atom site location: structure-invariant direct methods	Extinction coefficient: 0.018 (3)

### Special details

**Table:**

Geometry. Bond distances, angles *etc*. have been calculated using the rounded fractional coordinates. All su's are estimated from the variances of the (full) variance-covariance matrix. The cell e.s.d.'s are taken into account in the estimation of distances, angles and torsion angles
Refinement. Refinement on *F*^2^ for ALL reflections except those flagged by the user for potential systematic errors. Weighted *R*-factors *wR* and all goodnesses of fit *S* are based on *F*^2^, conventional *R*-factors *R* are based on *F*, with *F* set to zero for negative *F*^2^. The observed criterion of *F*^2^ > σ(*F*^2^) is used only for calculating -*R*-factor-obs *etc*. and is not relevant to the choice of reflections for refinement. *R*-factors based on *F*^2^ are statistically about twice as large as those based on *F*, and *R*-factors based on ALL data will be even larger.

### Fractional atomic coordinates and isotropic or equivalent isotropic displacement parameters (Å^2^)

**Table:**

	*x*	*y*	*z*	*U*_iso_*/*U*_eq_	
O8	0.2462 (4)	0.4170 (3)	0.8072 (2)	0.0736 (10)	
O9	0.0362 (6)	0.3831 (5)	0.6738 (2)	0.1186 (18)	
O11	0.1066 (3)	0.1532 (2)	0.82063 (14)	0.0429 (7)	
O19	0.0746 (4)	−0.2396 (2)	0.77708 (19)	0.0647 (9)	
N7	0.0831 (5)	0.3916 (3)	0.7666 (2)	0.0603 (11)	
N16	0.1731 (4)	−0.0929 (2)	0.67816 (18)	0.0445 (8)	
N20	0.1833 (4)	−0.0661 (2)	0.57957 (19)	0.0423 (8)	
C1	−0.2096 (5)	0.2889 (3)	0.9665 (3)	0.0561 (11)	
C2	−0.3242 (5)	0.3645 (4)	0.9620 (3)	0.0652 (14)	
C3	−0.3090 (5)	0.4456 (4)	0.8924 (3)	0.0649 (14)	
C4	−0.1778 (5)	0.4506 (3)	0.8273 (3)	0.0587 (11)	
C5	−0.0621 (4)	0.3744 (3)	0.8330 (2)	0.0469 (10)	
C6	−0.0729 (4)	0.2910 (3)	0.9021 (2)	0.0418 (9)	
C10	0.0452 (4)	0.2079 (3)	0.9105 (2)	0.0425 (9)	
C12	0.2155 (4)	0.0806 (3)	0.8501 (2)	0.0415 (9)	
C13	0.2212 (5)	0.0893 (3)	0.9544 (2)	0.0497 (11)	
C14	0.1110 (5)	0.1697 (3)	0.9934 (2)	0.0505 (11)	
C15	0.3075 (4)	0.0204 (3)	0.7665 (2)	0.0447 (10)	
C17	0.0708 (5)	−0.2192 (3)	0.6880 (2)	0.0472 (11)	
C18	−0.0432 (5)	−0.3260 (3)	0.5893 (3)	0.0592 (11)	
C21	0.3124 (4)	0.0530 (3)	0.5932 (2)	0.0414 (9)	
C22	0.4090 (4)	0.1216 (3)	0.7063 (2)	0.0502 (10)	
C23	0.3564 (4)	0.1109 (3)	0.5040 (2)	0.0440 (10)	
C24	0.2619 (4)	0.0440 (3)	0.4011 (3)	0.0487 (10)	
C25	0.3054 (5)	0.1030 (3)	0.3190 (3)	0.0539 (12)	
C26	0.4428 (5)	0.2314 (4)	0.3360 (3)	0.0556 (12)	
C27	0.5380 (5)	0.2980 (4)	0.4389 (3)	0.0628 (12)	
C28	0.4980 (5)	0.2405 (3)	0.5222 (3)	0.0565 (11)	
C29	0.4863 (6)	0.2965 (5)	0.2462 (4)	0.0812 (17)	
H1	−0.22270	0.23450	1.01380	0.0670*	
H2	−0.41350	0.36140	1.00620	0.0780*	
H3	−0.38780	0.49690	0.88980	0.0780*	
H4	−0.16700	0.50470	0.77980	0.0700*	
H13	0.28560	0.04970	0.99370	0.0600*	
H14	0.08830	0.19190	1.06270	0.0610*	
H15	0.39680	−0.01490	0.79860	0.0540*	
H18A	−0.08350	−0.41510	0.60300	0.0890*	
H18B	0.03140	−0.32780	0.53400	0.0890*	
H18C	−0.15080	−0.30380	0.56720	0.0890*	
H22A	0.39530	0.21130	0.73180	0.0600*	
H22B	0.54140	0.13360	0.71370	0.0600*	
H24	0.16790	−0.04200	0.38710	0.0580*	
H25	0.24060	0.05540	0.25040	0.0650*	
H27	0.63170	0.38400	0.45230	0.0750*	
H28	0.56480	0.28760	0.59050	0.0680*	
H29A	0.37170	0.28030	0.20040	0.1220*	
H29B	0.56460	0.25620	0.20630	0.1220*	
H29C	0.55040	0.39440	0.27470	0.1220*	

### Atomic displacement parameters (Å^2^)

**Table:**

	*U*^11^	*U*^22^	*U*^33^	*U*^12^	*U*^13^	*U*^23^
O8	0.0562 (17)	0.0598 (16)	0.093 (2)	0.0000 (13)	0.0194 (14)	0.0235 (14)
O9	0.146 (3)	0.173 (4)	0.052 (2)	0.063 (3)	0.025 (2)	0.045 (2)
O11	0.0469 (13)	0.0401 (11)	0.0322 (11)	0.0069 (9)	0.0038 (9)	0.0041 (9)
O19	0.0872 (19)	0.0492 (14)	0.0484 (15)	0.0091 (12)	0.0117 (12)	0.0156 (11)
N7	0.074 (2)	0.0525 (17)	0.0494 (18)	0.0117 (15)	0.0152 (15)	0.0165 (13)
N16	0.0507 (16)	0.0337 (13)	0.0352 (14)	0.0011 (11)	0.0061 (11)	0.0024 (10)
N20	0.0471 (15)	0.0354 (13)	0.0372 (14)	0.0060 (11)	0.0074 (11)	0.0073 (10)
C1	0.053 (2)	0.0454 (18)	0.058 (2)	0.0071 (16)	0.0147 (16)	0.0032 (15)
C2	0.046 (2)	0.055 (2)	0.076 (3)	0.0071 (17)	0.0142 (17)	−0.0045 (18)
C3	0.049 (2)	0.052 (2)	0.077 (3)	0.0146 (17)	−0.0064 (19)	−0.0037 (18)
C4	0.054 (2)	0.0459 (19)	0.062 (2)	0.0099 (16)	−0.0063 (17)	0.0042 (16)
C5	0.0431 (18)	0.0403 (16)	0.0397 (17)	0.0005 (14)	−0.0028 (13)	0.0006 (13)
C6	0.0390 (17)	0.0326 (15)	0.0387 (16)	0.0003 (12)	0.0022 (12)	−0.0003 (12)
C10	0.0443 (17)	0.0364 (15)	0.0343 (16)	0.0024 (13)	0.0076 (12)	0.0017 (12)
C12	0.0425 (17)	0.0342 (15)	0.0372 (16)	0.0050 (13)	−0.0002 (12)	0.0034 (12)
C13	0.061 (2)	0.0467 (18)	0.0371 (18)	0.0125 (16)	0.0043 (14)	0.0129 (14)
C14	0.065 (2)	0.0474 (18)	0.0332 (17)	0.0101 (16)	0.0105 (14)	0.0116 (13)
C15	0.0438 (18)	0.0418 (16)	0.0360 (17)	0.0050 (13)	−0.0009 (13)	0.0035 (13)
C17	0.056 (2)	0.0347 (16)	0.0447 (19)	0.0083 (14)	0.0122 (14)	0.0079 (13)
C18	0.067 (2)	0.0382 (17)	0.052 (2)	−0.0020 (16)	0.0078 (17)	0.0026 (15)
C21	0.0368 (16)	0.0359 (15)	0.0432 (17)	0.0043 (12)	0.0065 (12)	0.0066 (12)
C22	0.0432 (18)	0.0457 (17)	0.0453 (19)	−0.0002 (14)	0.0049 (13)	0.0037 (14)
C23	0.0371 (17)	0.0373 (15)	0.0501 (19)	0.0035 (13)	0.0059 (13)	0.0111 (13)
C24	0.0404 (17)	0.0403 (16)	0.052 (2)	0.0003 (13)	0.0050 (14)	0.0070 (14)
C25	0.050 (2)	0.054 (2)	0.048 (2)	0.0048 (16)	0.0060 (15)	0.0147 (15)
C26	0.045 (2)	0.057 (2)	0.064 (2)	0.0092 (16)	0.0070 (15)	0.0276 (17)
C27	0.048 (2)	0.052 (2)	0.072 (2)	−0.0072 (16)	0.0014 (17)	0.0230 (18)
C28	0.0463 (19)	0.0471 (18)	0.056 (2)	−0.0073 (15)	−0.0026 (15)	0.0128 (15)
C29	0.076 (3)	0.082 (3)	0.083 (3)	0.006 (2)	0.011 (2)	0.048 (2)

### Geometric parameters (Å, º)

**Table:**

O8—N7	1.229 (5)	C23—C28	1.406 (5)
O9—N7	1.213 (4)	C24—C25	1.379 (5)
O11—C10	1.376 (3)	C25—C26	1.385 (5)
O11—C12	1.381 (4)	C26—C27	1.384 (5)
O19—C17	1.236 (4)	C26—C29	1.507 (6)
N7—C5	1.461 (5)	C27—C28	1.380 (5)
N16—N20	1.390 (3)	C1—H1	0.9300
N16—C15	1.487 (4)	C2—H2	0.9300
N16—C17	1.361 (4)	C3—H3	0.9300
N20—C21	1.294 (4)	C4—H4	0.9300
C1—C2	1.362 (6)	C13—H13	0.9300
C1—C6	1.396 (5)	C14—H14	0.9300
C2—C3	1.378 (6)	C15—H15	0.9800
C3—C4	1.367 (6)	C18—H18A	0.9600
C4—C5	1.376 (5)	C18—H18B	0.9600
C5—C6	1.393 (4)	C18—H18C	0.9600
C6—C10	1.454 (4)	C22—H22A	0.9700
C10—C14	1.353 (4)	C22—H22B	0.9700
C12—C13	1.346 (4)	C24—H24	0.9300
C12—C15	1.480 (4)	C25—H25	0.9300
C13—C14	1.418 (5)	C27—H27	0.9300
C15—C22	1.531 (4)	C28—H28	0.9300
C17—C18	1.490 (5)	C29—H29A	0.9600
C21—C22	1.499 (4)	C29—H29B	0.9600
C21—C23	1.458 (4)	C29—H29C	0.9600
C23—C24	1.383 (5)		
			
C10—O11—C12	106.9 (2)	C27—C26—C29	121.3 (4)
O8—N7—O9	123.2 (4)	C26—C27—C28	121.9 (4)
O8—N7—C5	119.1 (3)	C23—C28—C27	120.1 (3)
O9—N7—C5	117.7 (4)	C2—C1—H1	119.00
N20—N16—C15	113.1 (2)	C6—C1—H1	119.00
N20—N16—C17	121.9 (2)	C1—C2—H2	120.00
C15—N16—C17	124.3 (2)	C3—C2—H2	120.00
N16—N20—C21	108.1 (2)	C2—C3—H3	120.00
C2—C1—C6	121.8 (3)	C4—C3—H3	120.00
C1—C2—C3	120.5 (4)	C3—C4—H4	120.00
C2—C3—C4	119.9 (4)	C5—C4—H4	120.00
C3—C4—C5	119.0 (3)	C12—C13—H13	126.00
N7—C5—C4	116.3 (3)	C14—C13—H13	126.00
N7—C5—C6	120.8 (3)	C10—C14—H14	127.00
C4—C5—C6	122.9 (3)	C13—C14—H14	127.00
C1—C6—C5	115.9 (3)	N16—C15—H15	109.00
C1—C6—C10	119.2 (3)	C12—C15—H15	109.00
C5—C6—C10	124.9 (3)	C22—C15—H15	109.00
O11—C10—C6	118.5 (2)	C17—C18—H18A	109.00
O11—C10—C14	109.5 (3)	C17—C18—H18B	109.00
C6—C10—C14	132.0 (3)	C17—C18—H18C	109.00
O11—C12—C13	109.3 (3)	H18A—C18—H18B	109.00
O11—C12—C15	116.1 (2)	H18A—C18—H18C	110.00
C13—C12—C15	134.5 (3)	H18B—C18—H18C	109.00
C12—C13—C14	107.5 (3)	C15—C22—H22A	111.00
C10—C14—C13	106.8 (2)	C15—C22—H22B	111.00
N16—C15—C12	113.3 (3)	C21—C22—H22A	111.00
N16—C15—C22	101.4 (2)	C21—C22—H22B	111.00
C12—C15—C22	114.1 (3)	H22A—C22—H22B	109.00
O19—C17—N16	119.2 (3)	C23—C24—H24	120.00
O19—C17—C18	123.3 (3)	C25—C24—H24	120.00
N16—C17—C18	117.5 (2)	C24—C25—H25	119.00
N20—C21—C22	114.0 (2)	C26—C25—H25	119.00
N20—C21—C23	121.3 (2)	C26—C27—H27	119.00
C22—C21—C23	124.7 (3)	C28—C27—H27	119.00
C15—C22—C21	103.4 (2)	C23—C28—H28	120.00
C21—C23—C24	122.5 (3)	C27—C28—H28	120.00
C21—C23—C28	119.5 (3)	C26—C29—H29A	109.00
C24—C23—C28	118.0 (3)	C26—C29—H29B	109.00
C23—C24—C25	120.9 (3)	C26—C29—H29C	109.00
C24—C25—C26	121.8 (3)	H29A—C29—H29B	109.00
C25—C26—C27	117.4 (4)	H29A—C29—H29C	109.00
C25—C26—C29	121.4 (4)	H29B—C29—H29C	110.00
			
C12—O11—C10—C6	179.6 (3)	C1—C6—C10—O11	147.6 (3)
C12—O11—C10—C14	−0.8 (3)	C1—C6—C10—C14	−31.9 (5)
C10—O11—C12—C13	0.2 (3)	C5—C6—C10—O11	−32.0 (4)
C10—O11—C12—C15	−176.0 (3)	C5—C6—C10—C14	148.5 (4)
O8—N7—C5—C4	129.4 (3)	O11—C10—C14—C13	1.0 (4)
O8—N7—C5—C6	−47.1 (4)	C6—C10—C14—C13	−179.5 (3)
O9—N7—C5—C4	−48.9 (5)	O11—C12—C13—C14	0.4 (4)
O9—N7—C5—C6	134.6 (4)	C15—C12—C13—C14	175.6 (3)
C15—N16—N20—C21	0.7 (4)	O11—C12—C15—N16	−67.3 (3)
C17—N16—N20—C21	−169.8 (3)	O11—C12—C15—C22	48.1 (4)
N20—N16—C15—C12	121.0 (3)	C13—C12—C15—N16	117.7 (4)
N20—N16—C15—C22	−1.7 (3)	C13—C12—C15—C22	−126.9 (4)
C17—N16—C15—C12	−68.8 (4)	C12—C13—C14—C10	−0.8 (4)
C17—N16—C15—C22	168.5 (3)	N16—C15—C22—C21	1.9 (3)
N20—N16—C17—O19	176.2 (3)	C12—C15—C22—C21	−120.3 (3)
N20—N16—C17—C18	−4.7 (5)	N20—C21—C22—C15	−1.8 (4)
C15—N16—C17—O19	6.8 (5)	C23—C21—C22—C15	178.7 (3)
C15—N16—C17—C18	−174.0 (3)	N20—C21—C23—C24	1.1 (5)
N16—N20—C21—C22	0.8 (4)	N20—C21—C23—C28	−179.9 (3)
N16—N20—C21—C23	−179.7 (3)	C22—C21—C23—C24	−179.4 (3)
C6—C1—C2—C3	0.4 (6)	C22—C21—C23—C28	−0.4 (5)
C2—C1—C6—C5	−0.3 (5)	C21—C23—C24—C25	179.0 (3)
C2—C1—C6—C10	−180.0 (3)	C28—C23—C24—C25	−0.1 (5)
C1—C2—C3—C4	−0.1 (6)	C21—C23—C28—C27	−178.5 (3)
C2—C3—C4—C5	−0.3 (6)	C24—C23—C28—C27	0.6 (5)
C3—C4—C5—N7	−176.0 (3)	C23—C24—C25—C26	−0.8 (6)
C3—C4—C5—C6	0.4 (5)	C24—C25—C26—C27	1.1 (6)
N7—C5—C6—C1	176.1 (3)	C24—C25—C26—C29	−178.6 (4)
N7—C5—C6—C10	−4.2 (4)	C25—C26—C27—C28	−0.5 (6)
C4—C5—C6—C1	−0.1 (4)	C29—C26—C27—C28	179.1 (4)
C4—C5—C6—C10	179.6 (3)	C26—C27—C28—C23	−0.3 (6)

### Hydrogen-bond geometry (Å, º)

Cg4 is the centroid of the C23–C28 ring.

**Table:**

*D*—H···*A*	*D*—H	H···*A*	*D*···*A*	*D*—H···*A*
C14—H14···O19^i^	0.93	2.56	3.462 (4)	162
C18—H18*C*···*Cg*4^ii^	0.96	2.69	3.595 (4)	158

Symmetry codes: (i) −*x*, −*y*, −*z*+2; (ii) −*x*, −*y*, −*z*+1.
